# PLK4 as a Key Regulator of Neuroblastoma Differentiation and a Promising Therapeutic Target

**DOI:** 10.7150/ijbs.111449

**Published:** 2025-07-28

**Authors:** Xiangdong Tian, Yuren Xia, Wenchen Gong, Kangwei Zhu, Yulong Yang, Zhiqiang Han, Yun Liu, Jie Li, Xin Li, Yuchao He, Mingyou Gao, Lu Chen, Hua Guo, Qiang Zhao

**Affiliations:** Tianjin Medical University Cancer Institute and Hospital, National Clinical Research Center for Cancer, Tianjin's Clinical Research Center for Cancer, Tianjin Key Laboratory of Digestive Cancer, Tianjin, China.

**Keywords:** differentiation therapy, PLK4, CXCR4, neuroblastoma, cyclin D1

## Abstract

**Background:** Neuroblastoma (NB) differentiation status critically influences prognosis and treatment response. Although differentiation therapy has shown clinical benefit, its efficacy remains limited. The molecular mechanisms driving NB differentiation are not fully understood. PLK4 has been linked to NB tumorigenesis, but its role in regulating differentiation remains unclear.

**Methods:** We investigated the role of PLK4 in neuroblastoma differentiation by modulating its expression both *in vitro* and *in vivo*. Through comprehensive analyses employing Western blotting, co-immunoprecipitation, immunofluorescence and murine neuroblastoma models, we identified downstream signaling pathways involved in PLK4-mediated regulation of neuronal genes. Pharmacological inhibition of PLK4 further confirmed its functional relevance in promoting neuroblastoma differentiation.

**Results:** PLK4 functions as a key regulator of neuroblastoma differentiation. Its depletion enhances neuronal maturation and sensitizes cells to 13-*cis* RA. Mechanistically, we identify a novel PLK4-CXCR4 signaling axis that governs neuroblastoma differentiation through PI3K/Akt-mediated modulation of cyclin D1 expression. The selective PLK4 inhibitor CFI-400945 exhibits dual anti-tumor activity by promoting terminal differentiation and suppressing proliferation.

**Conclusions:** Our study identifies PLK4 as a potential molecular switch governing NB differentiation and a promising therapeutic target to overcome resistance to 13-*cis* RA.

## Introduction

Dysdifferentiation is a hallmark of many human malignancies and has long been linked to oncogenesis and driver mutations^1^. This phenomenon is particularly prevalent in pediatric tumors such as neuroblastoma (NB), which arises from impaired differentiation of the sympathetic adrenal lineage^2^,^3^. As one of the most common solid tumors in children, NB accounts for nearly 10% of childhood cancer deaths^4^. The differentiation status significantly influences both the prognosis and the treatment response in NB^5^. While well-differentiated ganglioneuroma can be cured by surgical resection alone, poorly differentiated NB is associated with markedly reduced event-free survival, underscoring the inverse relationship between differentiation and clinical outcome. Although advances in intensive multimodal therapy-including induction chemotherapy, surgical resection, high-dose chemotherapy with autologous stem cell transplantation, radiotherapy, and maintenance with isotretinoin and anti-GD2 antibody-have improved overall survival, treating high-risk NB remains a major clinical challenge^5^,^6^. Nearly half of patients with high-risk disease experience relapse and ultimately succumb to progressive disease^3^.

Differentiation therapy exploits natural cellular pathways to push cancer cells toward benign or mature states. Unlike traditional treatments, it selectively targets malignant cells while sparing healthy tissues-minimizing toxicities like myelosuppression, a critical advantage for pediatric patients^7^,^8^. Deciphering differentiation mechanisms and optimizing differentiation-based therapies are crucial for halting tumor growth and enhancing survival. This strategy has proven transformative in acute promyelocytic leukemia (APL), achieving cure rates exceeding 95% and establishing a new standard of care^9^.

Unlike APL, where differentiation therapy achieves near-curative outcomes, its application in solid tumors such as NB remains significantly more challenging^9^,^10^. This is largely due to the complexity of NB biology, which involves heterogeneous cell populations and the activation of multiple, often redundant, oncogenic pathways that hinder differentiation. Despite these obstacles, differentiation therapy has been incorporated into standard maintenance regimens for high-risk NB patients^5^,^11^. The retinoic acid derivative 13-*cis* retinoic acid (13-*cis* RA) is currently the most widely used agent, aimed at promoting tumor cell maturation and preventing relapse after intensive therapy^10^,^12^,^13^. However, its clinical benefits are modest, improving event-free survival by only approximately 10%, with minimal impact on overall survival^14^,^15^. Moreover, differentiation therapy is frequently limited by adverse effects-including hyperlipidemia and mucositis-acquired resistance, unclear mechanisms of action, and a lack of more effective alternatives^16^. These limitations underscore the urgent need to better understand the regulatory landscape of NB differentiation and to identify more potent, selective, and well-tolerated differentiation therapies.

Polo-like kinases (PLKs) are serine/threonine kinases essential for cell cycle progression and mitosis^17^. However, the roles of PLKs in cell differentiation and underlying molecular mechanisms are not well understood. Among them, PLK4 is structurally distinct and functionally versatile, with established roles in promoting proliferation, invasion, and epithelial-mesenchymal transition (EMT) across multiple cancer types^18^,^19^. PLK4 is frequently overexpressed in various malignancies and has emerged as a promising therapeutic target^20^. We previously showed that PLK4 promotes EMT via PI3K/Akt signaling^21^, however, cancer-associated EMT is not simply a process of gaining migratory and invasive traits. NB differentiation is marked by morphological changes such as neurite outgrowth and filopodia formation, along with G0/G1 arrest and activation of lineage-specific markers^22^,^23^. Whether intermediate EMT states are defined as major determinants linked to dedifferentiation or simply represent a related but relatively independent relationship remains a topic of debate and requires further experimental validation. Elucidating the function of PLK4 in NB differentiation may reveal novel strategies for targeted differentiation therapy.

In this study, we identify PLK4 as a key regulator of NB differentiation. PLK4 expression is linked to axon development, and PLK4 knockdown promotes neuronal differentiation and enhances sensitivity to 13-*cis* RA. Mechanistically, PLK4 interacts with C-X-C chemokine receptor type 4 (CXCR4) to regulate cyclin D1 expression through the PI3K/Akt pathway, thereby modulating NB cell differentiation. We further show that PLK4 expression correlates not only with EMT but also with differentiation status, suggesting its potential as a biomarker to stratify patients with poor response to current therapies. Pharmacological inhibition of PLK4 reduces cell proliferation *in vitro* and suppresses tumor growth *in vivo*, supporting its therapeutic relevance. Together, these findings position PLK4 as a promising target in differentiation-based therapy for high-risk NB.

## Materials and Methods

***Cell lines.*** SK-N-Be(2), SH-SY5Y, SK-N-AS, and HEK293T cells were procured from American Type Culture Collection (USA), while SK-N-SH and IMR-32 cells were obtained from the Type Culture Collection of the Chinese Academy of Sciences (China). The identity of cell lines was authenticated by short tandem repeat analysis. The basal media used included Dulbecco's Modified Eagle's Medium/Nutrient Mixture F-12 for SK-N-Be(2), Dulbecco's Modified Eagle's Medium for SK-N-AS, SH-SY5Y, SK-N-SH, and HEK293T, and Minimum Essential Medium for IMR-32, supplemented with 1% penicillin/streptomycin and 10% fetal bovine serum (Corning). The cells were cultured in a 5% CO_2_ environment.

***Lentivirus production and cell transduction.*** HEK293T cells were transfected with the lentiviral vector and packaging plasmids (*VSVG* and* ∆R*) in a 2:1:1 ratio, using polyethylenimine (Polysciences, Warrington, USA). The viral supernatant was harvested 48 h post-transfection, filtered, and stored at -80°C. NB cells were transduced with generated virus in the presence of Polybrene (Solarbio, Beijing, China), performed as described previously^24^. Cells were then selected for 2-4 days with puromycin hydrochloride (0.5-1.5 μg/mL), then retreated monthly. Transfection of plasmids and siRNA into NB cells was performed using Lipofectamine 3000 (Invitrogen) following the manufacturer's protocol, ensuring high transfection efficiency with minimal cytotoxicity.

***Quantitative RT***-***PCR (qRT***-***PCR) and Western Blotting (WB).*** Total RNA was isolated from cells using Trizol reagent (Invitrogen), reverse-transcribed into cDNA using a qRT-PCR kit (Takara). The RNA was directly mixed with reagents supplied in the SYBR Green RT-PCR Kit (Vazyme), and subjected to one-step RT-PCR performed on a StepOnePlus™ Real-Time PCR System (Qiagen). Sequences of the primers for qRT-PCR are listed in *Table 1*. Western blotting were performed as described previously^24^. Briefly, WB were conducted using 50-80 μg of cell lysate per sample, separated on 8-10% polyacrylamide, 1-mm Bis-Tris Protein Gels (Thermo Fisher Scientific), and subsequently transferred to polyvinylidene difluoride membranes (Millipore). Detailed antibody information used were as follows: PLK4, Synaptophysin (SYN), MYCN, p-Akt^308^, p-Akt^473^, Akt, p-p38, p38, p-ERK, ERK and Cyclin D1 (1:1000, Cell Signaling Technology), H3, GAP43, PHOX2B and CXCR4 (1:1000, Abcam), E-cadherin (1:1000, BD Biosciences), N-cadherin (1:500, Santa Cruz Biotechnology). Membranes were scanned using a chemiluminescence imaging system (BLT Photon Technology). Protein quantification was performed using ImageJ, and statistical analysis was conducted using GraphPad prism 8.

***In Vitro Functional Assays.* 13-*cis RA treatment***: Twenty-four hours post in complete medium, cell lines were subjected to culture media containing 10μM 13-*cis* RA (Sigma-Aldrich) or 0.1% DMSO. Upon reaching 50-75% confluence, 100 cells were meticulously counted in triplicate and evaluated for neurite extension, characterized by neurite length ≥ soma length^22^. For RNA and protein analysis, the cells were cultured for 7 days and collected in phosphate-buffered saline. Total RNA and proteins were extracted as described earlier. Cell proliferation was determined at defined timepoints using the CCK-8 assay (Dojindo Laboratories). Each experiment was conducted with a minimum of three replicates. The cell cycle was analyzed on a FACS Aria flow cytometer (BD) after staining the cells with propidium iodide (BD Biosciences). The CellQuest software was used to acquire data as previously described, which were analyzed using FlowJo. ***Cell viability***: Cells were plated in a 96-well plate at a density of 5000 cells per well and exposed to RA at divergent concentrations for 48 h. And 10 μL of Cell Counting Kit-8 (CCK-8) reagent was added per well and incubated for 2-4 h according to the manufacturer's instructions. Absorbance measurements were acquired at a detection wavelength of 450 nm and a reference wavelength of 650 nm. Cell viability was calculated using the formula: Cell viability = [(As - Ab)/(Ac - Ab)] × 100%, where As, Ab, Ac represents the absorbance of the experimental well, the blank well and the control well, respectively. For ***Immunofluorescence**** (IF)* assays, cells were seeded onto 12-well chamber slides and treated as previously described. After removing the culture medium, cells were washed with phosphate-buffered saline (PBS) at room temperature, fixed in 4% paraformaldehyde, and permeabilized with 0.4% Triton X-100 containing 2% bovine serum albumin (BSA) for 1 hour at room temperature. Cells were then incubated overnight at 4°C with rabbit polyclonal anti-β-III tubulin antibody (1:100; Abcam) diluted in blocking buffer. After washing, cells were incubated in the dark with Alexa Fluor 594-conjugated goat anti-rabbit IgG (1:1000; Invitrogen) and counterstained with DAPI (1:5000; Sigma). Images were captured using a fluorescence microscope.

***Nuclear plasma separation experiment.*** After digestion, cells were collected by centrifugation and vigorously vortexed in CER I buffer (Thermo). Following incubation, CER II was added, and samples were vortexed and centrifuged. The resulting supernatant (cytoplasmic fraction) was collected. The pellet was then resuspended in ice-cold nuclear extraction reagent, vortexed, and centrifuged. The resulting supernatant (nuclear fraction) was collected and stored at -80°C for further use.

***Co-immunoprecipitation.*** Cell lysates were incubated with primary antibodies overnight at 4°C on a rotator. Protein-antibody complexes were captured using 30 µL of protein A/G Dynabeads (Life Technologies), with normal IgG used as a control. After centrifugation, the supernatants were subjected to SDS-PAGE and immunoblotting. Immunocomplexes were analyzed by western blot using the following antibodies: PLK4 (1:200, Abcam), CXCR4 (1:100, ABclonal), H3 (1:2000, Abcam), and GAPDH (1:500, Santa Cruz Biotechnology).

***Immunohistochemical staining.*** Immunohistochemistry (IHC) was performed to evaluate the expression of PLK4, CCND1, PHOX2B, CXCR4, and GAP43 in paraffin-embedded human neuroblastoma and mouse tumor tissues. As previously described, tissue sections were deparaffinized, rehydrated, and subjected to antigen retrieval using citrate buffer. Endogenous peroxidase activity was blocked with 3% hydrogen peroxide for 10 minutes. Sections were then incubated with primary antibodies for 30 minutes at room temperature, followed by overnight incubation at 4°C. After washing, sections were incubated with appropriate secondary antibodies for 1 hour at room temperature^21^,^24^. The primary antibodies used were as follows: PLK4 and PHOX2B (1:500; Abcam), CCND1 (1:400; Cell Signaling Technology), GAP43 (1:200; Cell Signaling Technology), and CXCR4 (1:500; ABclonal). Following DAB chromogenic detection and hematoxylin counterstaining, images were captured using a light microscope. Protein expression was quantified using the immunoreactive score (IRS), calculated by multiplying the staining intensity (score 0-3) by the percentage of positive cells (score 1-4).

***Fluorescent multiplex immunohistochemistry (mIHC).*** Prior to multiplex staining, the specificity of primary antibodies was validated by IHC using appropriate control tissues. As previously described, a multiplex immunofluorescence panel was established to assess differentiation status in 4-μm formalin-fixed, paraffin-embedded tissue sections^25^. Briefly, anti-PLK4 (1:250; Abcam) and anti-CCND1 (1:100; Cell Signaling Technology) antibodies were used as primary antibodies, with nuclei counterstained using DAPI. Opal Polymer HRP-conjugated anti-mouse and anti-rabbit secondary antibodies (Alphaxbio) were employed for signal amplification. Monoplex immunofluorescence and iterative multiplex staining were performed to optimize antibody-fluorophore pairing, fluorophore concentration, and staining sequence. Whole-slide images were acquired at multiple magnifications, and representative regions of interest were selected for multispectral imaging.

**Xenograft models. *Mouse xenograft tumor model****:* SK-N-AS cells (5 × 10⁶) suspended in Matrigel were subcutaneously injected into the groin area of 4-6-week-old female BALB/c nude mice (n = 5 per group). Mice were randomly assigned to receive either PLK4 knockdown cells or control cells. Tumor growth was monitored twice weekly using digital calipers. Mice were euthanized 6 weeks after injection. Tumor volume was calculated using the formula: V (mm³) = 0.5 × L × W², where L is the tumor length and W is the width. ***Patient-derived xenograft treatment***: NB diagnosis was confirmed by histopathological examination, and tumor staging was defined according to the International Neuroblastoma Staging System. Patient-derived NB tumor fragments were transplanted into SCID mice (see *Table 3* for clinical characteristics of the xenografts). Tumor growth was monitored regularly, and once the tumor volume reached approximately 200 mm³, mice were randomized into four treatment groups (n = 6 per group): (1) CTRL (vehicle control: 0.5-1% carboxymethylcellulose sodium [CMC-Na], oral gavage, once daily, 5 days per week); (2) 13-cis retinoic acid (13-*cis* RA, 40 mg/kg/day, oral gavage, 5 days per week); (3) CFI-400945 (2.5 mg/kg/day, dissolved in 0.5% CMC-Na, oral gavage, 5 days per week; Selleck); and (4) combination therapy (13-*cis* RA + CFI-400945). Tumor size was measured using digital calipers, and volume was calculated as previously described.

***Public datasets in this study.*** To increase statistical power, we incorporated publicly available RNA-sequencing data from a previous study (GSE49711; https://www.ncbi.nlm.nih.gov/geo/query/acc.cgi?acc=GSE49711). Gene set enrichment analysis (GSEA) was performed using the GSEA software (https://www.gsea-msigdb.org/gsea) to assess associations between PLK4 mRNA levels and biological pathways in neuroblastoma datasets. Sequencing data from NB tissues with different differentiation statuses have been deposited in the China National Center for Bioinformation (CNCB) under accession number PRJCA006532, and are publicly available at https://ngdc.cncb.ac.cn. CRISPR screening data were obtained from the DepMap database (version 24Q2, https://depmap.org/portal/). Copy-number Effect Removal by Estimating Scaling (CERES) gene effect scores for 34 NB cell lines were analyzed. All data used from the DepMap portal are publicly available^26^.

***Statistics.*** Statistical analyses were performed using SPSS 26.0. Pearson's χ² test was used to assess clinicopathologic correlations. Comparisons between groups were conducted using one- or two-way ANOVA, and two-group comparisons were performed using two-tailed Student's t-test. All statistical tests were two-sided, and p < 0.05 was considered statistically significant. Graphs were generated using GraphPad Prism 8. The significance levels are as follows: *p < 0.05, **p < 0.01, ***p < 0.001, ****p < 0.0001, and ns: no significance.

***Study approval.*** The Ethics Committee of Tianjin Medical University Cancer Institute and Hospital approved the study (Approval No. E20210664). The use of the patient-derived xenograft sample was conducted in accordance with ethical standards, and written and informed consent was obtained. The study adhered to the ethical guidelines outlined in the Helsinki Declaration. All animal experiments were approved by the Animal Ethics Committee of Tianjin Medical University Cancer Institute and Hospital (Approval No. KJJ-AM-2023015).

## Results

### The sensitivity of NB cell lines to 13-*cis* RA is correlated with the expression of PLK4

Neurite outgrowth is a well-established marker of NB cell differentiation *in vitro* and was quantified by calculating the ratio of neurite length to cell body diameter^9^,^27^. To explore the molecular mechanisms underlying NB growth and differentiation, we first analyzed genome-wide CRISPR-CERES screening data from the DepMap database and identified 599 genes with strong dependency (Copy-number Effect Removal by Estimating Scaling, CERES < -1) in ≥80% of NB cell lines. We then performed transcriptomic profiling comparing five well-differentiated and five poorly differentiated NB tumors, which revealed over 470 significantly dysregulated genes (raw *p* < 0.01, |log₂FC| > 3). Twenty genes overlapped between these datasets, suggesting potential roles in driving NB differentiation. Among them, PLK4 was the only member of the PLK family (Supplementary figure 1A). We previously demonstrated that elevated PLK4 expression is an independent predictor of poor prognosis in NB^21^, a finding supported by data from the TARGET and GSE49711 cohorts (Supplementary figure 1B). Based on these observations, we further investigated the role of PLK4 in NB differentiation.

**Figure 1:**
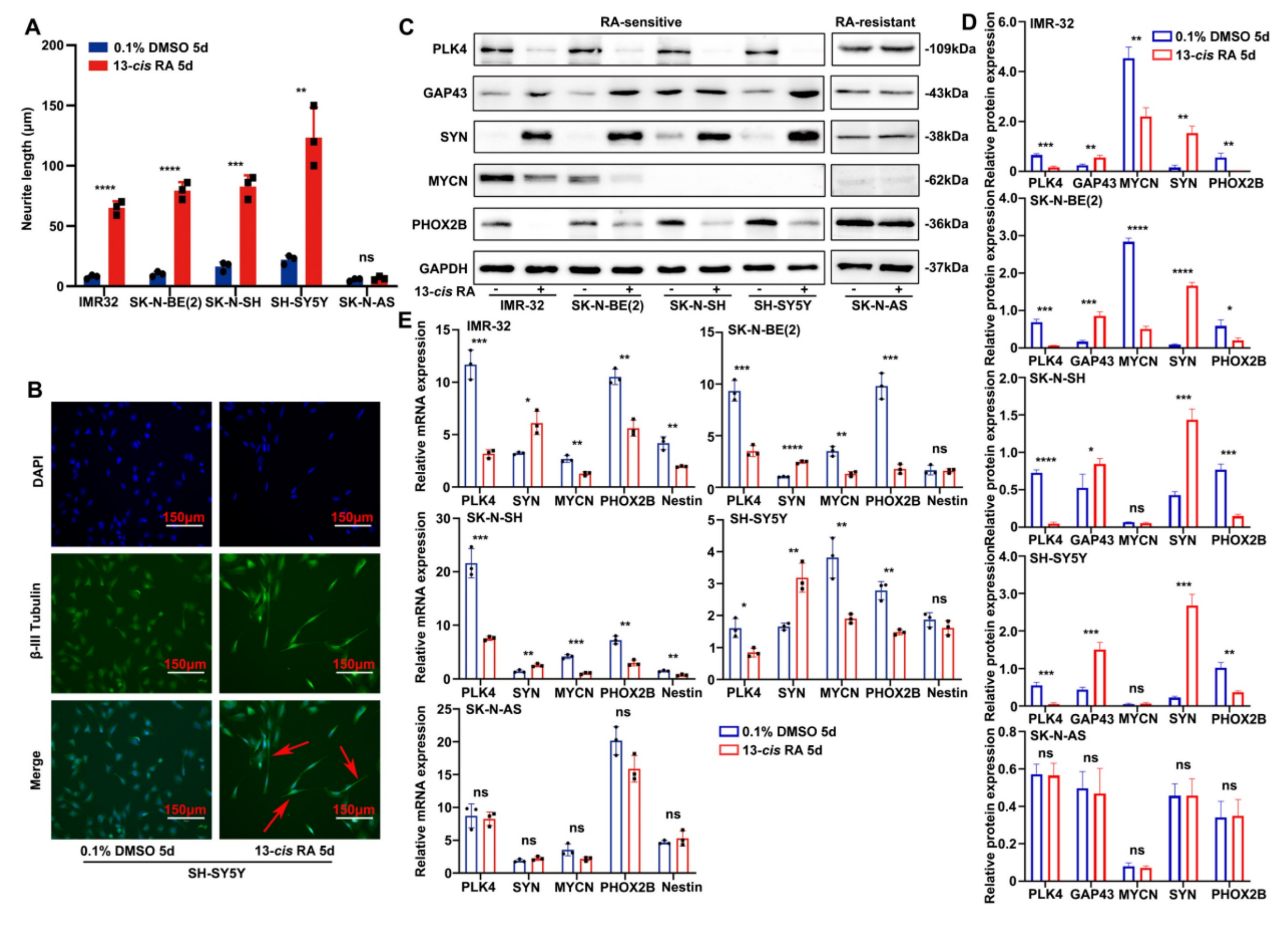
** The sensitivity of NB cell lines to 13-*cis* RA is correlated with the expression of PLK4. A**. Neurite extension assessment by measuring neurite length to cell body diameter in neuroblastoma cells treated with 0.1% DMSO or 10 μM 13-*cis* RA for 5 days. **B**. Immunofluorescence staining showing β-III tubulin expression and neurite outgrowth in SH-SY5Y cells treated with DMSO or 13-*cis* RA. **C**. Western blotting analysis of PLK4, SYN, MYCN, PHOX2B and GAP43 expression in 5 NB cell lines. **D**. Quantification of relative protein expression was performed, and data represent mean ± SEM from 3-4 independent experiments. **E**. Relative expressions of PLK4 and differentiation-associated markers were measured by quantitative reverse transcription PCR (RT-PCR). *p < 0.05, **p < 0.01, ***p < 0.001, ****p < 0.0001, ns: no significance.

Similarly as our previous studies, E-cadherin and N-cadherin expression in tumor tissues varied with PLK4 levels and correlated with the degree of differentiation (Supplementary figure 1C). However, in the RA-sensitive SH-SY5Y cell line, PLK4 overexpression altered E-cadherin and N-cadherin expression independently of RA dosage within a defined range (Supplementary figure 1D). We hypothesize that, beyond its role in EMT, PLK4 may contribute to NB oncogenesis through additional mechanisms, including the regulation of differentiation. To explore this possibility, we analyzed mRNA expression profiles from the GSE49711 dataset and found that gene sets associated with neuronal differentiation and development were significantly enriched in NB tumors with low PLK4 expression (Supplementary figure 1E). To assess the sensitivity of NB cell lines to 13-*cis* RA, cells were treated with either 0.1% dimethyl sulfoxide (DMSO, control) or 10 μM RA for 5 days. RA insensitivity was defined by the absence of morphological changes, such as neurite extension or cell body elongation, following treatment. Notably, RA-sensitive cell lines-including IMR-32, SK-N-BE(2), SK-N-SH, and SH-SY5Y-exhibited pronounced morphological changes, including robust neurite outgrowth. In contrast, the RA-insensitive line SK-N-AS showed no such differentiation features (Figure 1A and Supplementary figure 1F). To confirm RA-induced neuronal differentiation, we assessed the expression of class β-III tubulin-a neuron-specific marker-by immunofluorescence in SH-SY5Y cells. RA-treated cells exhibited marked neurite extension compared with the control group, supporting successful neuronal differentiation (Figure 1B and Supplementary figure 1G).

We quantified PLK4 expression in NB cell lines following RA treatment using both WB and reverse-transcription polymerase chain reaction (RT-PCR) (Figure 1C-E). In RA-sensitive cells, 13-*cis* RA treatment led to a marked downregulation of PLK4, MYCN, and the neural transcription factor PHOX2B, alongside upregulation of synaptic markers such as synaptophysin (SYN), growth-associated protein 43 (GAP43), and neuron-specific enolase. In contrast, RA-insensitive cell lines showed no significant changes in PLK4 or differentiation marker expression.

### PLK4 influences the differentiation of NB cells and PLK4-knockdown sensitizes them to the effects of 13-*cis* RA

**Figure 2:**
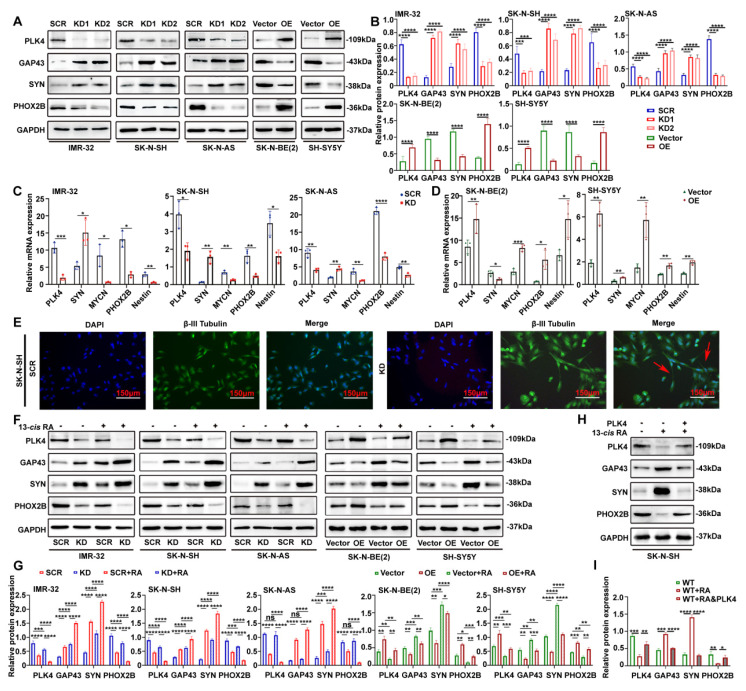
** PLK4 influences the differentiation of NB cells and PLK4-knockdown sensitizes them to the effects of 13-*cis* RA. A**. PLK4 and differentiation-associated markers expression levels were assessed using Western blotting in NB cell lines (KD1: PLK4-knockdown1, KD2: PLK4-knockdown2, OE: PLK4 overexpression). **B**. Quantification of PLK4 and differentiation-associated markers expression was performed, and data represent mean ± SEM from 3-4 independent experiments. **C & D**. Expression levels of PLK4 and differentiation-associated markers were evaluated using RT-PCR in NB cell lines. **E**. Immunofluorescence of β-III Tubulin in SK-N-SH cells showing neurite outgrowths (red arrows) seen following PLK4-knockdown. **F**. Western blotting analysis of PLK4 and differentiation-associated markers protein levels in NB cell lines following PLK4-knockdown (the term KD as used in the text and figure pertains to KD2). **G**. Quantification of relative protein expression in 2G. Data were presented as mean ± SEM. n = 3-4. **H**. Rescue experiments assessing whether RA-induced differentiation is reversed by PLK4 overexpression in SK-N-SH. **I**. Quantification of above protein expression in 2H. *p < 0.05, **p < 0.01, ***p < 0.001, ****p < 0.0001, ns: no significance.

We analyzed PLK4 expression across five neuroblastoma (NB) cell lines with varying malignant potential: MYCN-amplified, RA-sensitive SK-N-BE(2) and IMR-32; MYCN-non-amplified, RA-sensitive SK-N-SH and SH-SY5Y; and MYCN-non-amplified, RA-insensitive SK-N-AS. PLK4 was highly expressed in IMR-32, SK-N-SH, and SK-N-AS, but comparatively lower in SK-N-BE(2) and SH-SY5Y (Supplementary figure 1H). Using lentiviral vectors, we achieved PLK4 knockdown in IMR-32, SK-N-SH, and SK-N-AS, and overexpression in SK-N-BE(2) and SH-SY5Y. These manipulations were validated through both WB (Figure 2A&B) and RT-PCR (Figure 2C&D). Unless otherwise specified, knockdown refers to KD2 throughout the text and figures.

RNA sequencing (RNA-seq) of PLK4-knockdown cells (three replicates per group) revealed significant transcriptional changes. Gene Ontology (GO) analysis indicated enrichment of pathways related to cell differentiation (Supplementary figure 2A). Consistent with findings from the GSE49711 dataset, gene sets associated with neural development and regulation were upregulated in PLK4-knockdown cells (Supplementary figure 2B).

PLK4 knockdown promoted neuronal differentiation, as evidenced by the notably increased expression of SYN, GAP43 and decreased expression of PHOX2B, whereas PLK4 overexpression yielded the opposite results (Figure 2A-D). PLK4-knockdown in NB cells led to the formation of neurites and cell body elongation (Figure 2E). While wild-type SK-N-AS cells do not differentiate into neuron-like cells in response to RA, PLK4-knockdown SK-N-AS cells displayed profound neurite outgrowth (Supplementary figure 2C).

Differentiation involves cell cycle arrest and the implementation of gene programs^21^. Flow cytometry revealed that PLK4 knockdown would cause cells to accumulate in the G0/G1 phase, along with a decrease in the number of cells in the other cell cycle phases, indicating significant differences or a tendency of cell cycle arrest in the G0/G1 phase (Supplementary figure 2D). PLK4 expression and NB cell proliferation were closely associated according to the sequencing data. Cell Counting Kit-8 (CCK-8) assays demonstrated a substantial reduction in the viability of IMR-32, SK-N-SH, and SK-N-AS cells after shRNA-mediated knockdown of PLK4 (Supplementary figure 2E). These results align with a diminished proliferative phenotype and the upregulation of antiproliferative characteristics. Conversely, PLK4 overexpression heightened the proliferative capacity of NB cells, accompanied by a notable decrease in the proportion of cells in the G0/G1 phase (Supplementary figure 2D&E).

Given that PLK4 directly influences NB cell differentiation, we next examined whether the effects of 13-*cis* RA on differentiation were mediated through PLK4 regulation. Silencing of PLK4 led to increased expression of differentiation markers such as SYN and GAP43, and decreased expression of PHOX2B, across both MYCN-amplified and non-amplified NB cell lines. These effects were further enhanced when PLK4-knockdown, RA-sensitive cells were treated with 13-*cis* RA. Conversely, PLK4 overexpression promoted a de-differentiated phenotype, underscoring its role in maintaining an undifferentiated, tumorigenic state regardless of MYCN status (Figure 2F&G). To further assess whether RA-induced differentiation is mediated by PLK4 downregulation, we performed rescue experiments in RA-sensitive SK-N-SH cells. Treatment with 13-*cis* RA for 5 days decreased PHOX2B and increased GAP43 and SYN expression, indicative of neuronal differentiation. Notably, PLK4 overexpression for 48 hours attenuated these changes, reinforcing its role as a negative regulator of RA-driven differentiation (Figure 2H&I). Together, these findings highlight PLK4 as a critical regulator of NB cell differentiation.

### The PI3K/Akt pathway plays a crucial role in PLK4-mediated differentiation

**Figure 3:**
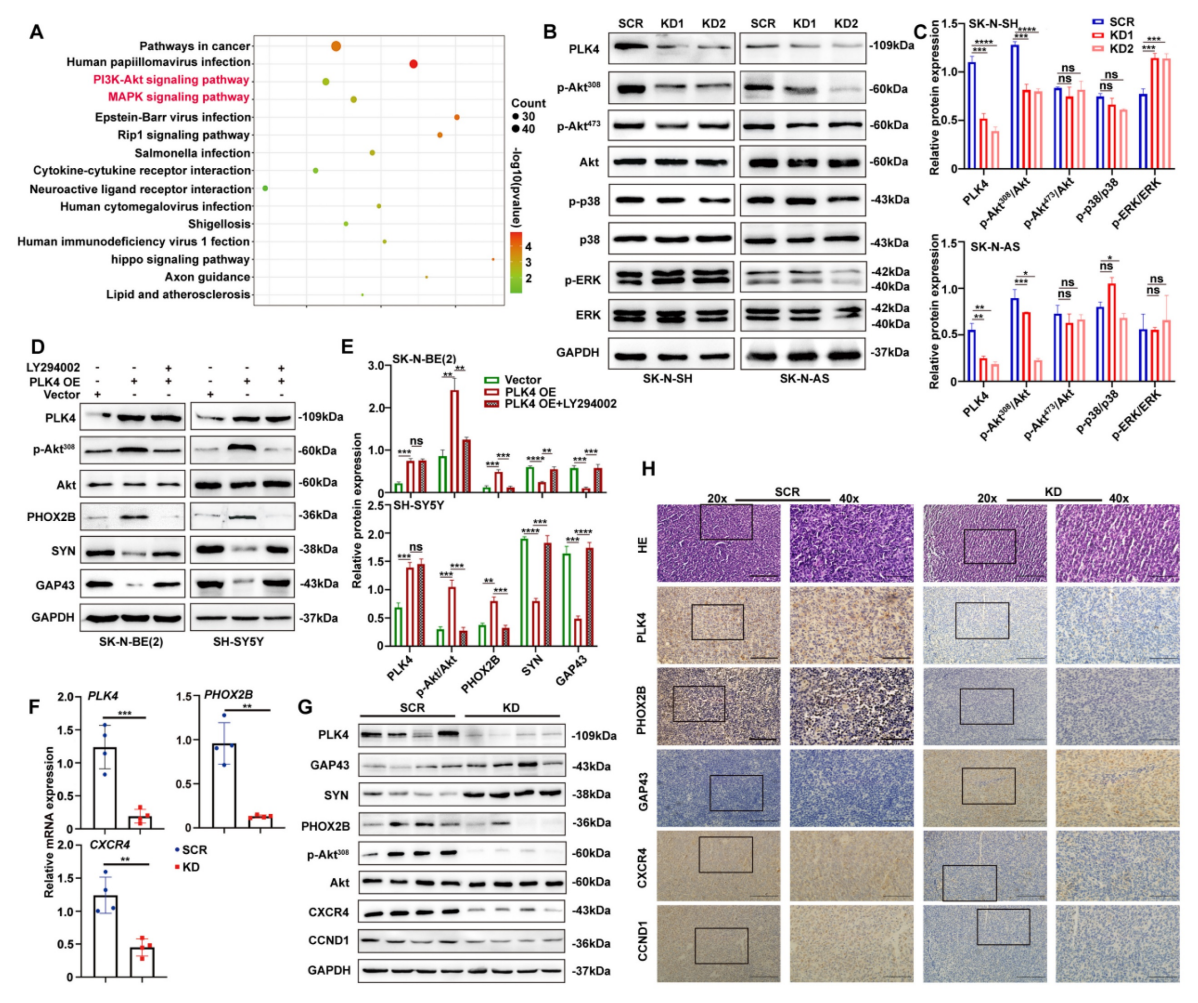
** The PI3K/Akt pathway plays a crucial role in PLK4-mediated differentiation. A**. Results of a pathway analysis obtained from RNA sequencing (RNA-seq) in the PLK4-knockdown group (|Log2 fold change| > 1 and adjusted *p* value < 0.05). **B**. Western blotting analysis demonstrating the effect on different signaling pathway activation following PLK4-knockdown in SK-N-SH and SK-N-AS. **C**. Protein expression levels of various signaling pathways were quantified. Data represent mean ± SEM from 3-4 independent experiments. **D**. PLK4-overexpression NB cells were pretreated with LY294002. Western blotting analysis of the protein levels of p-Akt^308^, Akt, and differentiation-associated protein. **E**. Quantification of above protein expression in 3D. The mRNA (**F**) and protein (**G**) levels of PLK4, differentiation protein and Akt signaling pathway components assessed in murine tumor tissues by RT-PCR and Western blotting. **H**. IHC analysis of PLK4 and differentiation protein expressions in two groups. (KD1: PLK4-knockdown1, KD2: PLK4-knockdown2, OE: PLK4 overexpression). *p < 0.05, **p < 0.01, ***p < 0.001, ****p < 0.0001, ns: no significance.

Transcriptomic analysis identified 244 differentially expressed genes following PLK4 knockdown, including 31 upregulated and 213 downregulated genes. Pathway enrichment analysis revealed significant involvement of the PI3K/Akt and MAPK signalling pathways (Figure 3A; see *Table 2* for enriched pathways).

Akt signalling is activated in 50-60% of primary NB samples and is strongly associated with poor prognosis^28^. To investigate downstream mechanisms, we examined the PI3K/Akt and MAPK pathways by WB. In PLK4-knockdown NB cell lines, phosphorylation of Akt at Thr308 was markedly reduced, while phosphorylation at Ser473 remained unchanged (Figure 3B&C and Supplementary figure 3A). We next treated PLK4-overexpressing NB cells with or without pathway-specific inhibitors-LY294002 (PI3K/Akt, 5 μM), BIRB796 (p38 MAPK, 20 μM), and PD98059 (MEK/ERK, 100 μM)-for 48 hours. PLK4 overexpression increased p-Akt^308^ levels, but this increase was markedly blunted upon incubating the cells with the respective inhibitors. Notably, LY294002 markedly suppressed the PLK4-induced expression of differentiation markers (Figure 3D&E), whereas BIRB796 and PD98059 had only minor effects (Supplementary figure 3B).

We sought to verify these *in vitro* findings in an *in vivo* model with RA-insensitive SK-N-AS cells. The mouse tumor xenograft model was established subcutaneously implanting with either PLK4 knockdown or the control SK-N-AS cells into the groins each nude mouse. Same as our previous results, both tumor volume and weight were notably reduced in the PLK4-knockdown group compared with the control group (Supplementary figure 3C-E). Consistent with the *in vitro* results, PLK4 expression was markedly reduced in the PLK4-knockdown xenografts. This was accompanied by upregulation of GAP43 and SYN, and downregulation of p-Akt^308^ and PHOX2B compared with the control group (Figure 3F&G). Immunohistochemistry (IHC) experiments further confirmed these results, in agreement with WB and RT-PCR analyses (Figure 3H). Together, these data indicate that PLK4 suppresses differentiation, and PLK4 knockdown markedly promotes NB cell differentiation both *in vitro* and *in vivo*.

### PLK4 mediates cell differentiation by interacting with CXCR4

**Figure 4:**
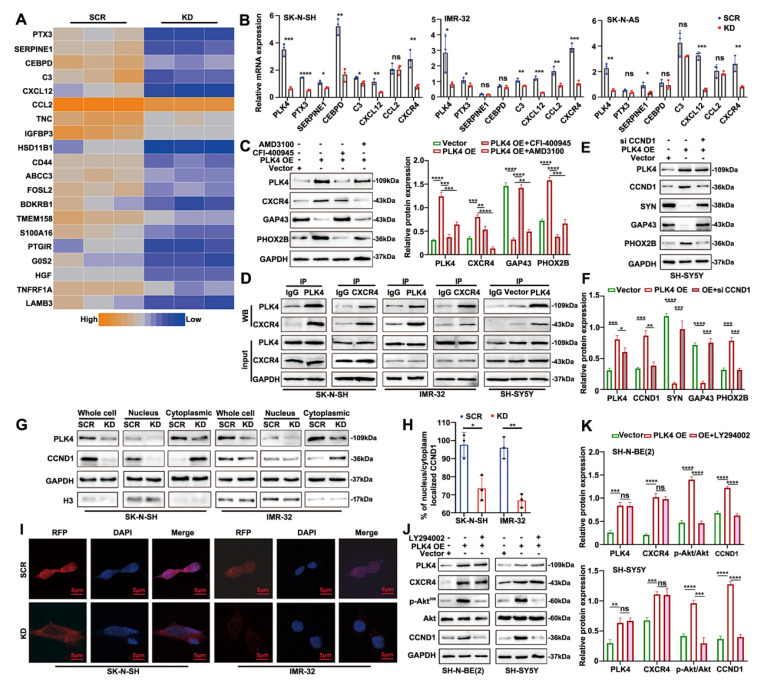
** PLK4 mediates cell differentiation by interacting with CXCR4. A**. Heatmap showing differentially expressed genes in PLK4-knockdown cells compared with the control cells. n = 3. **B**. Relative mRNA levels were of top differentially expressed genes were detected using RT-PCR. **C**. As shown in the left panel, Western blotting detected the expression of PLK4, CXCR4 and differentiation-associated markers after SH-SY5Y being treated with CFI-400945 or AMD-3100. The right panel corresponds to the quantification of relative protein expression. Data were presented as mean ± SEM. n = 3. **D**. Co-IP of endogenous as well as exogenous PLK4 interacts with CXCR4 in NB cells. **E**. PLK4-overexpression cells were treated with or without CCND1 siRNA, PLK4, CCND1 and differentiation-associated markers was examined. **F**. Quantification of relative protein expression in 3E. Data were presented as mean ± SEM. n = 3. Nuclear-cytoplasmic fractionation experiments (**G**) and Immunofluorescence (**H**-**I**) showed that PLK4 knockdown reduced the nuclear localization of cyclin D1. **J**. PLK4-overexpression cells were infected with LY294002 (5 μM, 48 h). The protein level of p-Akt and CCND1 were demonstrated by Western blotting. **K**. Quantification of relative protein expression in 3J (KD: PLK4-knockdown2, OE: PLK4 overexpression). *p < 0.05, **p < 0.01, ***p < 0.001, ****p < 0.0001, ns: no significance.

To explore the molecular mechanisms by which PLK4 regulates neuronal genes, we sought to identify its potential interaction partners. We focused on the top 20 differentially expressed genes in PLK4-knockdown cells compared with controls. Transcripts with a standard deviation >1 were log-transformed, mean-centered, and visualized by heatmap, with expression levels represented from low (blue) to high (orange) (Figure 4A). Among the top 20 differentially expressed transcripts, C-X-C motif chemokine ligand 12 (CXCL12) stood out, given its well-established roles in neurodevelopment and cell migration, particularly in organ-specific metastasis. RT-PCR further confirmed differential mRNA expression of both CXCL12 and its receptor CXCR4 between PLK4-knockdown and control groups (Figure 4B). Notably, CXCL12 is the only confirmed ligand for CXCR4, a key receptor broadly expressed across multiple tumor types. C-X-C chemokine receptor type 4 (CXCR4) signaling regulates tumor cell proliferation, bone marrow metastasis, and resistance to chemotherapy^29^. NB is characterized by early bone marrow metastasis, and the degree of cellular differentiation is closely associated with metastatic potential^30^. Given that the CXCR4-CXCL12 axis mediates tumor cell homing to the bone marrow and promotes metastasis in vivo^31^, we hypothesized that PLK4 may influence NB cell differentiation through its interaction with CXCR4.

To elucidate the relationship between PLK4 and CXCR4 in NB differentiation, we overexpressed CXCR4 in PLK4-knockdown cells and treated PLK4-overexpressing cells with the CXCR4 inhibitor AMD3100. CXCR4 protein levels were reduced upon PLK4 knockdown and elevated with PLK4 overexpression, suggesting a regulatory link between PLK4 and CXCR4. While CXCR4 overexpression and AMD3100 treatment induced only modest changes in PLK4 levels, both interventions led to marked alterations in the expression of differentiation-associated markers (Supplementary figure 3F). Furthermore, in PLK4-overexpressing SH-SY5Y cells, treatment with either the PLK4 inhibitor CFI-400945 or AMD3100 resulted in decreased expression of both PLK4 and CXCR4. Notably, CFI-400945 elicited more pronounced changes in differentiation markers compared to AMD3100, suggesting that PLK4 may modulate NB cell differentiation through interaction with CXCR4 (Figure 4C). Co-immunoprecipitation and reciprocal co-immunoprecipitation in SK-N-SH and IMR-32 cells confirmed a physiological interaction between endogenous PLK4 and CXCR4. Furthermore, PLK4 overexpression in SH-SY5Y cells enhanced PLK4-CXCR4 binding (Figure 4D).

As shown above, PLK4 knockdown in NB cells led to a marked reduction in CXCR4 mRNA and protein levels, implicating PLK4 as a positive regulator of CXCR4 expression. Therefore, we hypothesized that PLK4 might influence CXCR4 protein stability. To test this, we monitored CXCR4 turnover following treatment with cycloheximide (CHX, 10 μg/mL), a protein synthesis inhibitor. CXCR4 degradation progressively increased with extended CHX exposure, whereas PLK4 overexpression attenuated this effect, suggesting a stabilizing role for PLK4 (Supplementary figure 3G). Furthermore, treatment with the proteasome inhibitor MG132 (2 μM) restored CXCR4 levels in PLK4-overexpressing cells (Supplementary figure 3H), supporting a model in which PLK4 modulates CXCR4 degradation via a proteasome-dependent mechanism.

Cyclin D1, encoded by CCND1 on chromosome 11q13, is a key regulator of the G1 phase of the cell cycle. Overexpression of CCND1 promotes cell cycle progression and tumorigenesis, while alterations in its expression or nuclear localization can disrupt the G1-S phase transition^32^. In this study, CCND1 expression was reduced in PLK4-knockdown cells compared to controls *in vitro* (Figure 3G&H)*.* Silencing CCND1 in PLK4-overexpressing cells altered the expression of CCND1 and differentiation-associated markers, without significantly affecting PLK4 levels (Figure 4E&F). Analysis of the GSE49711 dataset revealed a positive correlation between PLK4 and both *CCND1* and *PHOX2B*, and a negative correlation with *GAP43* and *SYN* expression (Supplementary figure 3I&J). Given that cyclin D1 participates in neuronal differentiation and is regulated upstream by CXCR4^33^, we hypothesized that PLK4 may interact with CXCR4 to modulate cyclin D1 expression or nuclear localization, thereby influencing NB cell differentiation. Nuclear-cytoplasmic fractionation showed reduced nuclear localization of cyclin D1 upon PLK4 knockdown (Figure 4G), which was further confirmed by immunofluorescence (Figure 4H&I).

To investigate the mechanism by which PLK4 regulates cyclin D1 expression, PLK4-overexpressing cells were treated with or without LY294002 (5 μM) for 48 h. LY294002 suppressed p-Akt^308^ levels without notably affecting PLK4 or CXCR4 expression, but significantly suppressed cyclin D1 expression (Figure 4J&K). Together, these findings support a model in which the PLK4-CXCR4 axis regulates CCND1 via the PI3K/Akt pathway, thereby modulating NB cell differentiation.

### PLK4 strongly correlates with the differentiation capacity and clinical outcome of NB

**Figure 5:**
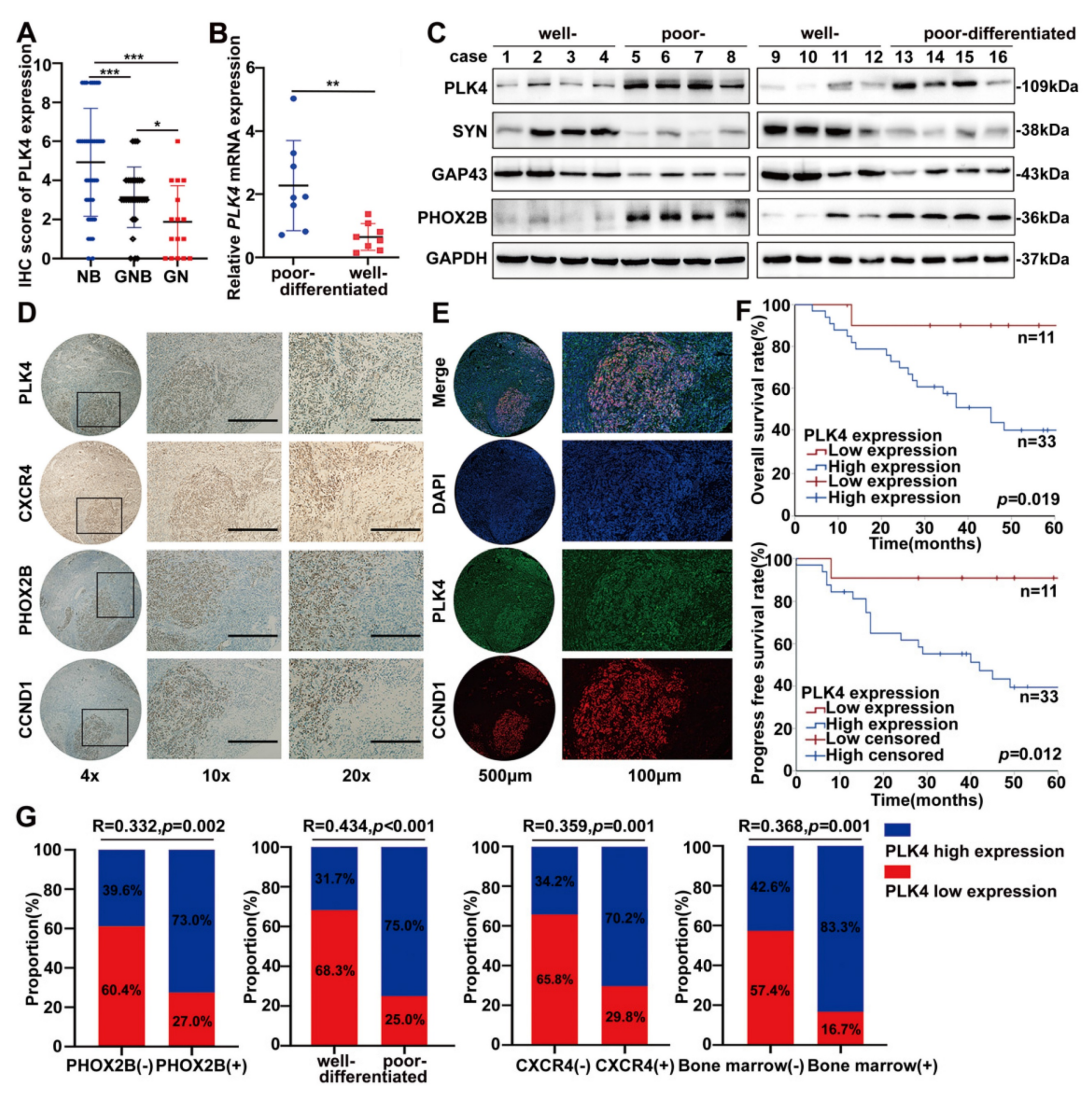
** PLK4 expression strongly correlates with the differentiation capacity and clinical outcome of NB. A**. Quantification of IHC score (NB neuroblastoma; GNB ganglioneuroblastoma; GN ganglioneuroma). RT-PCR (**B**) and Western blotting (**C**) analysis of PLK4 expression in NB tissues with different differentiation status. **D**. Assessment of PLK4, PHOX2B, CXCR4, and cyclin D1 expression levels and their associations in the same tumor tissues. **E**. Intratumoral heterogeneity was confirmed through fluorescent multiplex immunohistochemistry. **F**. The Kaplan-Meier survival analysis of overall survival and progress free survival for NB patients in poor-differentiated subgroup. **G**. Correlation analysis between PLK4 expression and PHOX2B expression, differentiation status, CXCR4 expression and bone marrow metastasis. *p < 0.05, **p < 0.01, ***p < 0.001.

The correlation between PLK4 expression and the clinicopathological parameters of NB was assessed through IHC staining (Supplementary figure 4A). Based on IHC scores, a tissue microarray analysis of 85 patient samples revealed high PLK4 expression in 46 cases and low expression in 39. Elevated PLK4 levels were associated with poorer overall and progress free survival, consistent with our previous findings (Supplementary figure 4B). Remarkably, PLK4 expression varied significantly across tumor types, with the highest levels observed in neuroblastoma, followed by ganglioneuroblastoma and ganglioneuroma (Figure 5A). Poor-differentiated NB tumors were strongly correlated with decreased survival (Supplementary figure 4C). Consistently, PLK4 expression was markedly elevated in NB samples, particularly in poor-differentiated and advanced-stage tumors, as evidenced by WB and RT-PCR (Figure 5B&C and Supplementary figure 4D&E). Additionally, consistent with data from GSE49711, PLK4 protein expression was positively correlated with the differentiation-associated marker PHOX2B while negatively correlated with SYN and GAP43 (Supplementary figure 3J, Figure 5C). These findings identify PLK4 as a potential prognostic indicator and a marker of differentiation status in NB.

Differentiation status strongly influences NB prognosis, yet reliable biomarkers reflecting differentiation and treatment sensitivity remain limited. We assessed PLK4, PHOX2B, CXCR4, and cyclin D1 expression by IHC in tumor tissues from the same patients exhibiting variable differentiation status. PLK4 expression in the tumor tissues markedly varied with the degree of differentiation, and it was notably elevated in poor-differentiated regions compared with well-differentiated regions, wherein the expression of PHOX2B, CXCR4, and cyclin D1 was higher (Figure 5D). Intratumoral heterogeneity was confirmed through fluorescent multiplex immunohistochemistry, which indicated diminished PLK4 expression in well-differentiated regions. Furthermore, the activity of cyclin D1 were attenuated, which confirmed our hypothesis that cyclin D1, regulated by PLK4, plays key roles during NB cell differentiation (Figure 5E).

Notably, PLK4 low expression emerged as a critical protective factor influencing the prognosis of the high-risk subgroup of patients with poor-differentiated NB (Figure 5F). And based on IHC staining scores and Spearman's rank correlation analysis, PLK4 expression was negatively correlated with the degree of differentiation while it was positively correlated with PHOX2B and CXCR4 expression, bone marrow metastasis, and INSS staging (Figure 5G and Supplementary figure 4F). Therefore, PLK4 is strongly associated with the differentiation status of NB, being predominantly highly expressed in poor-differentiated NBs. This makes PLK4 a viable drug target for differentiation therapy.

### Inhibiting PLK4 can suppress the proliferation of NB cells and promote differentiation

Next, we explored whether NB progression could be mitigated through the pharmacological disruption of PLK4. The orally available PLK4 inhibitor CFI-400945, when used in monotherapy, has demonstrated promising efficacy in inhibiting the growth of solid tumors *in vivo* without exhibiting any discernible toxicity.

**Figure 6:**
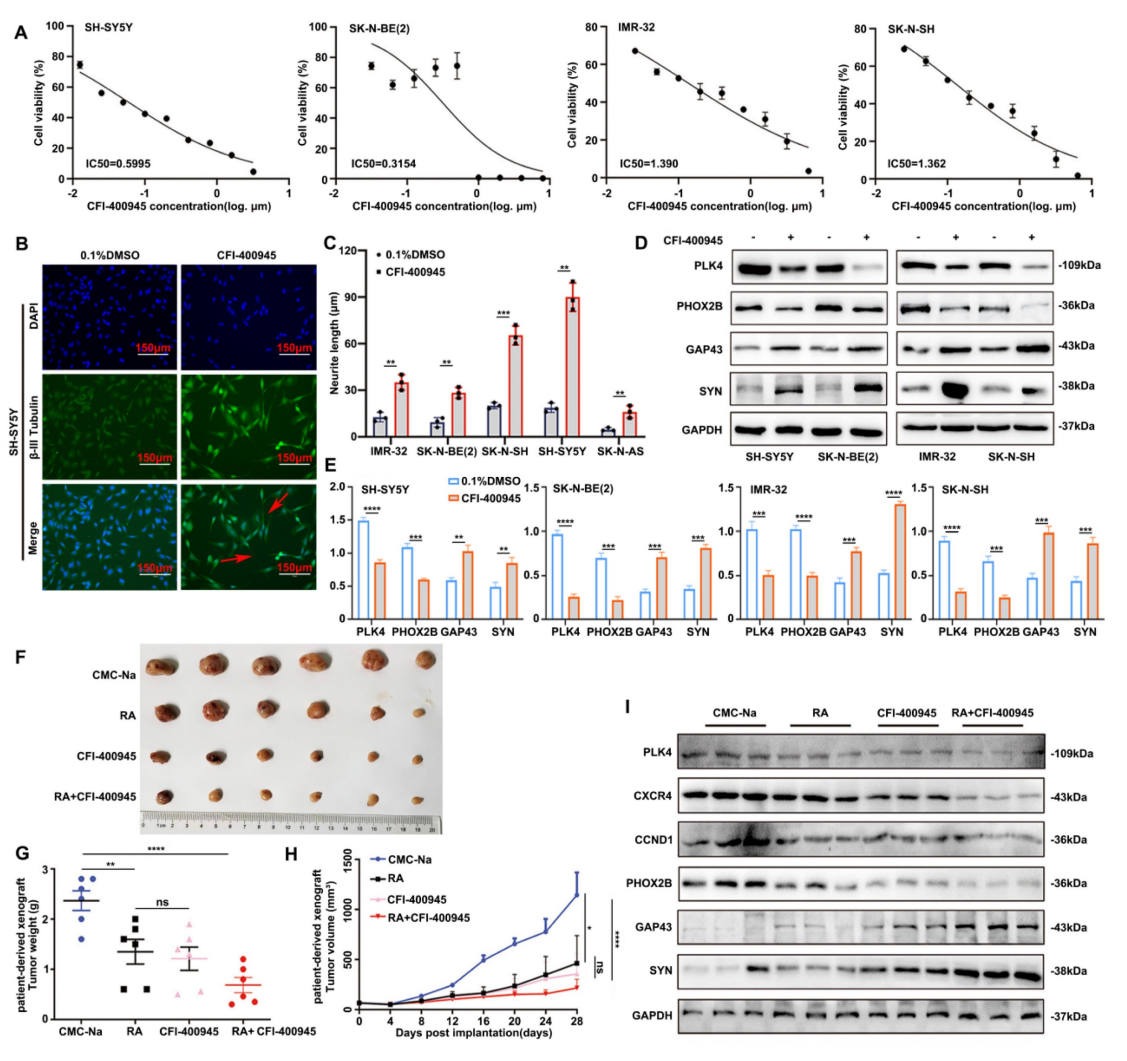
** Inhibiting PLK4 can suppress the proliferation of NB cells and promote differentiation. A**. IC50 values of CFI-400945 treatment in neuroblastoma cell lines for 72 h. IC50 was calculated in GraphPad Prism 8.0 and displayed respectively. **B**. Immunofluorescence of β-III Tubulin in SH-SY5Y cells treated with CFI-400945 or 0.1% DMSO for 5 days.** C**. Neurite extension assessment in neuroblastoma cells treated with CFI-400945 or 0.1% DMSO for 5 days. Treatment with CFI-400945 resulted in a significant increase in neurite outgrowth in NB cell lines, indicating differentiation of surviving cells. Data reported as mean ± SEM. **D**. Western blotting analysis of cells after treatment with CFI-400945 compared with the control cells. **E**. Quantification of relative protein expression in 6D. Data were presented as mean ± SEM. n = 3. **F-G**. Tumor development in SCID mice and tumor weights of tumors obtained from xenografts were assessed. **H**. Tumor volume in the all groups. **I**. The inhibition efficiency of PLK4, CXCR4 and the expression of differentiation-associated marker were examined by Western blotting. *p < 0.05, **p < 0.01, ***p < 0.001, ****p < 0.0001, ns: no significance.

The NB cell lines were significantly responsive to CFI-400945, as shown by the results of the 3-(4,5-dimethylthiazol-2-yl)-2,5-diphenyltetrazolium bromide assay. The half-maximal inhibitory concentration (IC50) values varied between 0.3154 μM and 1.390 μM, with IMR-32 exhibiting the highest IC50 (Figure 6A). Concentrations below the calculated IC50 values were deliberately chosen for the subsequent experiments to evaluate early morphological alterations at nonlethal doses of the drug. NB cell lines showed enhanced neurite outgrowth after 72 h of exposure to CFI-400945, indicating the differentiation of surviving cells (Figure 6B&C, Supplementary figure 4G&H). Moreover, the expression of differentiation-related markers discernibly changed in all the cell lines (Figure 6D&E). These findings underscore the heterogeneous sensitivity of NB cells to PLK4 inhibition, confirming that the effects of CFI-400945 on cell differentiation were not cell line-specific.

The impact of CFI-400945 on tumor growth *in vivo* was validated using PLK4-mutant patient-derived xenograft models. The combination of CFI-400945 and RA significantly inhibited tumor growth and lowered tumor weight in mice, whereas no significant disparity was detected in terms of tumor growth and reduced tumor weight between the RA and CFI-400945 groups (Figure 6F-H). WB revealed that PLK4 was most significantly downregulated in the RA + CFI-400945 treatment group. The expression of CXCR4 as well as CCND1 was also significantly attenuated in this group, and the expression levels of PHOX2B and GAP43 were notably altered (Figure 6I). Furthermore, *in vivo* experiments confirmed that CFI-400945 promotes the differentiation of NB cells and inhibits the proliferation of tumor cells.

## Discussion

The acquisition of increased cellular phenotypic plasticity allows cells to escape terminal differentiation and plays a central role in cancer development^34^. This plasticity-including de-differentiation, trans-differentiation, and termination of differentiation-drives malignant transformation and contributes to therapy resistance^34^,^35^. NBs are highly heterogeneous, ranging from spontaneously regressing tumors to poorly differentiated, metastatic disease^36^. Unlike in APL, differentiation therapy has shown limited efficacy in high-risk neuroblastoma. This limitation is partly attributed to MYCN amplification, which promotes an undifferentiated, proliferative phenotype and correlates with poor prognosis^37^,^38^. Although MYCN downregulation is essential for neuronal maturation, direct targeting remains difficult due to delivery challenges and lack of effective inhibitors^39^,^40^. A deeper understanding of the mechanisms underlying differentiation and resistance to retinoid-based therapy is critical for identifying novel biomarkers and improving treatment outcomes.

**Figure 7:**
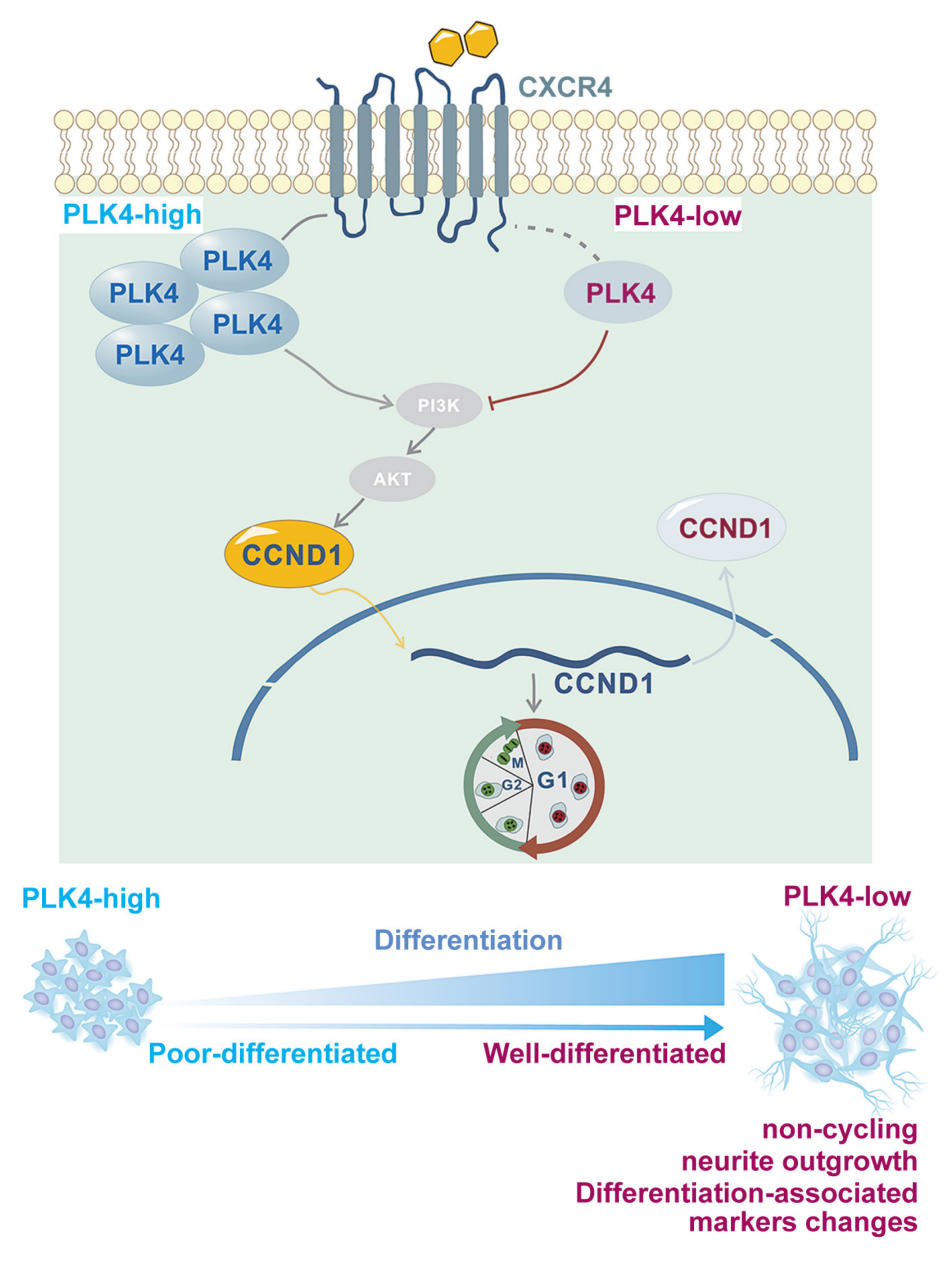
** Working model**.

Neuronal cell proliferation and differentiation are important processes that govern cell fate^41^. Under normal conditions, differentiation typically occurs after cell cycle exit^42^,^43^. Disruption of this process often marks the onset of rapid malignant proliferation^44^. Previous studies have shown that PLK4 inhibition suppresses cell division and enhances immune surveillance in aggressive hepatocellular carcinoma, further supporting its role in regulating the cell cycle and DNA content^45^. In this study, both *in vitro* and* in vivo* experiments demonstrated that PLK4 depletion suppressed proliferation, induced cell cycle arrest, enhanced differentiation in synergy with RA, and restored NB cell sensitivity to RA. PLK4 was a key driver of proliferation and facilitated the shift from differentiation to de-differentiation (Figure 7). PLK4 expression correlated with prognosis and differentiation status in clinical NB samples. Pharmacological inhibition of PLK4 promoted differentiation to some extent, underscoring its potential as a therapeutic target in high-risk NB.

Accumulating evidence indicates that multiple factors collectively shape the differentiation process and final cell fate of tumor cells^46^,^47^. Both EMT and differentiation involve significant morphological changes^48^, making it challenging to distinguish the differentiation phenotype from that of EMT. We previously reported that PLK4-driven EMT promotes cell proliferation and motility. Notably, cancer-associated EMT is not simply a process of gaining migratory and invasive traits. Instead, it represents a highly plastic program essential for both physiological and pathological contexts, involving broad cellular reprogramming-including metabolic shifts, epigenetic changes, and dedifferentiation^48^,^49^. In the current study, E-cadherin and N-cadherin expression in tumor tissues varied to some extent with the degree of differentiation. However, within a certain dosage range, it seems that changes in RA dosage did not affect E-cadherin and N-cadherin expression. Therefore, whether intermediate EMT states are defined as major determinants linked to dedifferentiation or simply represent a related but relatively independent relationship remains a topic of debate and requires further experimental validation, and we speculate that the role of PLK4 in NB is multifaceted.

Neuronal differentiation is intimately coordinated with the cell cycle and is accompanied by changes in the expression of differentiation-related markers, furthermore, cell cycle exit represented the fundamental step to trigger cell differentiation^41^,^42^. G0/G1 cell cycle arrest represents a reversible state. In certain cellular states, such as senescence, dormancy, and differentiation, the cell cycle gets arrested at the G1/S transition. Senescence is a gradual and irreversible process marked by functional decline^50^. Dormancy involves cells transitioning between a proliferative state and a slower-cycling, invasive state^51^. Meanwhile, cell differentiation is characterized by the expression of specific genes and functional changes^52^, which were confirmed in this study. G1 phase-regulating genes significantly contribute to NB tumorigenesis and the maintenance of an undifferentiated phenotype^53^. We observed that PLK4 knockdown resulted in an accumulation of cells in the G1 phase, along with a concurrent decrease in cell numbers in the G2/M and S phases, implicating PLK4 in the facilitation of the G1/S transition in NB cells. Cyclin D1 possesses the capability to abbreviate the G1 phase and drive the transition to the S phase in response to mitogenic signals^54^. It plays a crucial role, which gradually diminishes as neural progenitors progress towards terminal neurons^55^. Elevated levels of cyclin D1 may potentially impair NB cell differentiation^56^. The nuclear overexpression of cyclin D1 may contribute to neuronal differentiation, which is indicative of its role as a downstream effector of other regulating genes or as a driver that hinders NB differentiation^56^,^57^. The direct effects of PLK4 expression on cyclin D1 expression in NB cells had not been conclusively demonstrated before this study.

Using RT-PCR, we showed that the expressions of PLK4 and CXCL12 were positively correlated. Since CXCR4 is a crucial receptor for CXCL12^58^ and the CXCR4-CXCL12 pathway is involved in NB cell growth^33^, we also assessed the relative expression of CXCR4 in NB cells. CXCR4 expression is correlated with the stage of the disease and bone marrow metastasis^59^. The pivotal role of the CXCR4-CXCL12 axis in NB is reflected in its strong association with migration towards the bone marrow compartment^33^,^60^. As a regulators of neuronal migration, CXCR4, localized close to the cell membrane, can interact with the intracellular cytoskeleton in migrating neurons throughout the development of the central nervous system^61^. However, impact of the interaction between CXCR4 and other intracellular proteins on neuron development is unclear. In this study, IHC staining revealed that PLK4 expression was positively correlated with CXCR4 expression and bone marrow metastasis in a subset of NB samples. CXCR4 inhibition could prevent tumor growth and reduce tumor cell survival by downregulating target genes like *CCND1*^33^. We discovered that PLK4 knockdown was associated with the downregulation of CXCR4 and CCND1, whereas PLK4 overexpression was associated with their upregulation. Immunofluorescence and nuclear-cytoplasmic fractionation assays confirmed that PLK4 overexpression enhanced the recruitment of cyclin D1 to the nucleus, while PLK4 knockdown increased the localization of cyclin D1 to the cytoplasm. These findings suggest that PLK4 influences NB cell differentiation by modulating cyclin D1 abundance as well as activity via CXCR4 (Figure 7).

Differentiation is triggered by the activation of specific pathways in normal cells, typically coupled with a halt in proliferation^62^. Activation of Akt has been detected in 50-60% of all primary NB samples^28^. Ying *et al.* uncovered a novel connection between Akt inhibition and NB cell differentiation^63^. In this study, an Akt inhibitor promoted PLK4-induced differentiation, which further underlines the importance of Akt signaling in the progression of NB. The depth of our findings could be enhanced by conducting comparative studies involving a PLK4 inhibitor, a CXCR4 inhibitor, and an Akt inhibitor to explore their respective impacts on differentiation. In addition, as anticancer agents, they exhibit varying degrees of pro-apoptotic functionality^45^,^64^. Equally important, it is crucial in practical clinical applications to optimize the stimulation of differentiation and facilitation of apoptosis prioritizing safety. Acknowledging these limitations, such comparative analyses would provide a more comprehensive understanding in future investigations and manifest an attractive anticancer strategy.

In higher eukaryotes, two pivotal events mark the initiation of proliferation: centrosome duplication and DNA replication^65^. The orchestration of centrosome duplication is highly intricate throughout the cell cycle, ensuring precise spindle assembly and proper cell division^66^. As reported, a synthetic lethal relationship exists between PLK4 and TRIM37, the latter of which is encoded on chromosome 17q. At the core of centriole duplication lies the evolutionarily conserved PLK4, the mitotic crisis due to centriole dysregulation upon PLK4 inhibition when TRIM37 is overexpressed^67^. This mechanism may contribute to NB differentiation, the current study do not include centriole quantification, although further experiments are needed to explore this hypothesis. Maintenance of the cell cycle and the prevention of differentiation contribute to oncogenesis, whereas differentiation initiates as cells exit the cell cycle. Promoting the differentiation of poorly differentiated cells into mature cells presents an alternative avenue for clinical intervention. In comparison to traditional cancer treatments, the application of differentiation therapy to treat malignant tumors is still in its nascent stages. Another limiting factor is the absence of universally recognized diagnostic targets or markers for inducing differentiation. As maintenance therapy for high-risk neuroblastoma with minimal residual disease, 13-*cis* RA acts through retinoic acid receptors and diverse pathways, but its limited specificity, efficacy, and resistance highlight the need for alternative strategies^14^,^16^. Our findings suggest that dysregulated PLK4 contributes to NB tumorigenesis and impaired differentiation. While PLK4 inhibition promotes differentiation, the inhibitor CFI-400945 also targets Aurora kinases, complicating interpretation and offering no clear in vivo benefit over 13-*cis* RA. Besides, future studies will employ more selective chemical and genetic tools to confirm on-target effects, and assess therapeutic relevance in advanced models such as organoids and clinical samples. Further work should also clarify PLK4's molecular mechanisms, particularly its interaction with CXCR4, and evaluate selective inhibitors alone or in combination therapies. Overall, future investigations should explore PLK4's therapeutic potential beyond EMT, with a particular focus on its role in driving NB differentiation.

## Supplementary Material

Supplementary figures.

## Figures and Tables

**Table 1:** Primer sequences in this manuscript

Primer	Forward	Reverse
*PLK4*	GCATCTCAAGAATATGTGAAA	TTTCACATATTCTTGAGATGC
*PHOX2B*	CCTGAAGATCGACCTCACAGAG	TTTTGCCCGAGGAGCCGTTCTT
*MYCN*	ACCACAAGGCCCTCAGTACCTC	TGACAGCCTTGGTGTTGGAGGA
*NESTIN*	ACTGGAGTCTGTGGAAGTGA	TCAGCTCCCGCAGCAGACTCACC
*PTX3*	CGAAATAGACAATGGACTCCATCC	CTCATCTGCGAGTTCTCCAGCA
*SERPINE1*	CTCATCAGCCACTGGAAAGGCA	GACTCGTGAAGTCAGCCTGAAAC
*C3*	GTGGAAATCCGAGCCGTTCTCT	GATGGTTACGGTCTGCTGGTGA
*CCL2*	AGAATCACCAGCAGCAAGTGTCC	TCCTGAACCCACTTCTGCTTGG
*CXCR12*	CTCAACACTCCAAACTGTGCCC	CTCCAGGTACTCCTGAATCCAC
*CXCR4*	TCACCAAGTTTGGACACCGT	GCCACACACTTGTGGAGCTA
*β-actin*	TCATCACCATTGGCAATGAG	CACTGTGTTGGCGTACAGGT

**Table 2:** Signaling pathways through the identification of differentially expressed genes based on RNA sequencing

Term	Count	Count%	*p*-value	FDR	Fold Enrichment
hsa05200: Pathways in cancer	48	4.968944	1.45E-04	0.007682	1.75848474
hsa05165: Human papillomavirus infection	37	3.830228	1.35E-05	0.001788	2.17453107
hsa04151: PI3K-Akt signaling pathway	31	3.20911	0.004453535	0.051312	1.70353209
hsa04010: MAPK signaling pathway	28	2.898551	0.002269414	0.037587	1.85268928
hsa05169: Epstein-Barr virus infection	25	2.587992	9.75E-05	0.007682	2.40757889
hsa04015: Rap1 signaling pathway	25	2.587992	1.79E-04	0.007905	2.3158616
hsa05132: Salmonella infection	25	2.587992	0.002088988	0.037587	1.95313629
hsa04060: Cytokine-cytokine receptor interaction	25	2.587992	0.01660764	0.106027	1.64857944
hsa04080: Neuroactive ligand-receptor interaction	25	2.587992	0.095004708	0.286094	1.37770803
hsa05163: Human cytomegalovirus infection	23	2.380952	0.002635446	0.039046	1.98855316
hsa05131: Shigellosis	23	2.380952	0.00798429	0.070528	1.81143506
hsa05170: Human immunodeficiency virus 1 infection	22	2.277433	0.002799537	0.039046	2.01873218
hsa04390: Hippo signaling pathway	21	2.173913	1.43E-04	0.007682	2.60202539
hsa04360: Axon guidance	21	2.173913	0.001006093	0.029624	2.24460432
hsa05417: Lipid and atherosclerosis	21	2.173913	0.007047123	0.065855	1.90008365

**Table 3:** Clinical characteristics of patient-derived xenograft

	Case 1	Case 2
Age	23 months	42 months
INSS	IV	IV
Primary tumor site	adrenal gland	retroperitoneal
Genetics	MYCN amplified	MYCN non-amplified

INSS: International Neuroblastoma Staging System

## Abbreviations

NB: neuroblastoma
APL: acute promyelocytic leukemia
13-c*is* RA: 13-cis retinoic acid
PLKs: Polo-like kinases
EMT: epithelial-mesenchymal transition
IF: immunofluorescence
WB: western blotting
qRT-PCR: quantitative RT-PCR
PBS: phosphate-buffered saline
BSA: bovine serum albumin
CCK-8: cell counting Kit-8
IHC: immunohistochemistry
IRS: immunoreactive score
IHC: fluorescent multiplex immunohistochemistry
GSEA: gene set enrichment analysis
CNCB: China National Center for Bioinformation
CERES: copy-number effect removal by estimating scaling
RNA-seq: RNA sequencing
GO: gene ontology
CXCL12: C-X-C motif chemokine ligand 12
IC50: half-maximal inhibitory concentration
