# Supplementary material for: PLK4 as a Key Regulator of Neuroblastoma Differentiation and a Promising Therapeutic Target
Source: Int J Biol Sci. 2025 Jul 28;21(11):4979–96. doi: 10.7150/ijbs.111449 (PMC12374821; doi:10.7150/ijbs.111449)
Supplement: Supplementary file 1 — Supplementary figures. [file ijbsv21p4979s1.zip › Supplementary figures/supplementary figure legends.pdf]

Supplementary figure 1. A. Venn diagram showing the overlap between essential genes and differentiation-associated genes in neuroblastoma. **B**. Kaplan-Meier curves of overall neuroblastoma survival probability between PLK4 high group and low group generated from publicly available neuroblastoma data sets accessible at the TARGET and GSE49711 analysis. **C.** Western blotting analysis of PLK4, E-Cadherin and N-Cadherin expression in NB tissues with different differentiation status. **D**. Western blotting analysis of E-Cadherin and N-Cadherin expression in PLK4-overexpression SH-SY5Y with different dosages of 13-*cis* RA. **E**. GSEA analysis indicates enrichment and upregulation of genes associated with neurogenesis, neuron development and neuron differentiation in samples with low expression levels of PLK4. **F**. Photomicrographs depict five NB cells following a 5-day treatment with either 10 μM 13-*cis* RA or 0.1% DMSO. The presented images are representative of three independent samples. **G**. Immunofluorescence staining showing neurite outgrowth in SH-SY5Y cells with DMSO or 13-*cis* RA (Scale bar, 5 μm). **H**. Western blotting analysis of PLK4 expression in 5 NB cell lines.

Supplementary figure 2. A. GO analysis was conducted on differentially expressed genes subsequent to the knockdown of PLK4. n = 3, Fisher's exact test. **B**. GSEA plot of axon extension, axongenesis, axon guidance, neuron development, neurogenesis, regulation of neurogenesis between the PLK KD group and control group. **C**. Representative photomicrographs for IMR-32, SK-N-SH and SK-N-AS cells showing neurite outgrowths (red arrows) after PLK knockdown. **D**. Cell cycle distribution analysis following PLK4 KD/OE. Data represent mean ± SD. Three technical replicates shown. **E**. Significant changes in proliferation of NB cell lines following PLK4 KD/OE. $^{**}p < 0.01$, $^{***}p < 0.001$, $^{****}p < 0.0001$, ns: no significance.

Supplementary figure 3. A. Western blotting analysis demonstrating the effect on different signaling pathway activation following PLK4-knockdown in IMR-32. **B**. PLK4-overexpression SK-N-BE(2) and SH-SY5Y cells were pretreated with PD98059 or BIRB796. Western blotting analysis of the protein levels of pathway and differentiation-associated protein. **C**-**D**. Tumor development in mice post injection of SK-N-AS cells stably infected with shRNA against the SCR or KD. Tumor volumes were measured every 4 days. Data are presented as the mean ± SEM of tumors in 5 mice. **E**. Tumor weights in the two groups. **F**. CXCR4 modulation in PLK4-altered cells influences differentiation marker expression, indicating a regulatory link in NB differentiation. **G**. CXCR4 protein levels were assessed by Western blotting in Vector and PLK4 OE cells treated with 10 μg/mL cycloheximide (CHX) for the indicated time points. **H**. CXCR4 protein levels were evaluated in Vector and PLK4 OE cells with or without treatment with the proteasome inhibitor MG132 (2 μM). Correlation between PLK4 expression and CCND1(**I**), GAP43, PHOX2B as well as SYN (**J**) in samples obtained from the GSE49711 dataset. *$p < 0.05$, **$p < 0.01$, ***$p < 0.001$, ****$p < 0.0001$.

Supplementary figure 4. A. The expression of PLK4 in tissue microarray (TMA) samples was evaluated by immunohistochemistry. **B**. Kaplan-Meier plots displayed overall survival (left) and progress free survival (right) that were associated with PLK4 expression levels. **C**. Kaplan-Meier curve displaying overall survival and progress free survival for patients with different differentiation status. Western blotting (**D**) and RT–PCR (**E**) analysis of PLK4 expression in NB tissue with different stage. **F**. Correlation analysis between PLK4 expression and International Neuroblastoma Staging System (INSS) staging. **G**. Photomicrographs depict five NB cells following a 5-day treatment with either CFI-400945

or 0.1% DMSO. **H**. Immunofluorescence staining showing β-III tubulin expression and neurite outgrowth in SH-SY5Y cells treated with DMSO or CFI-400945 (Scale bar, 5 μm). ***$p < 0.001$.
