# Supplementary figures and images for: PLK4 as a Key Regulator of Neuroblastoma Differentiation and a Promising Therapeutic Target
Source: Int J Biol Sci. 2025 Jul 28;21(11):4979–96. doi: 10.7150/ijbs.111449 (PMC12374821; doi:10.7150/ijbs.111449)

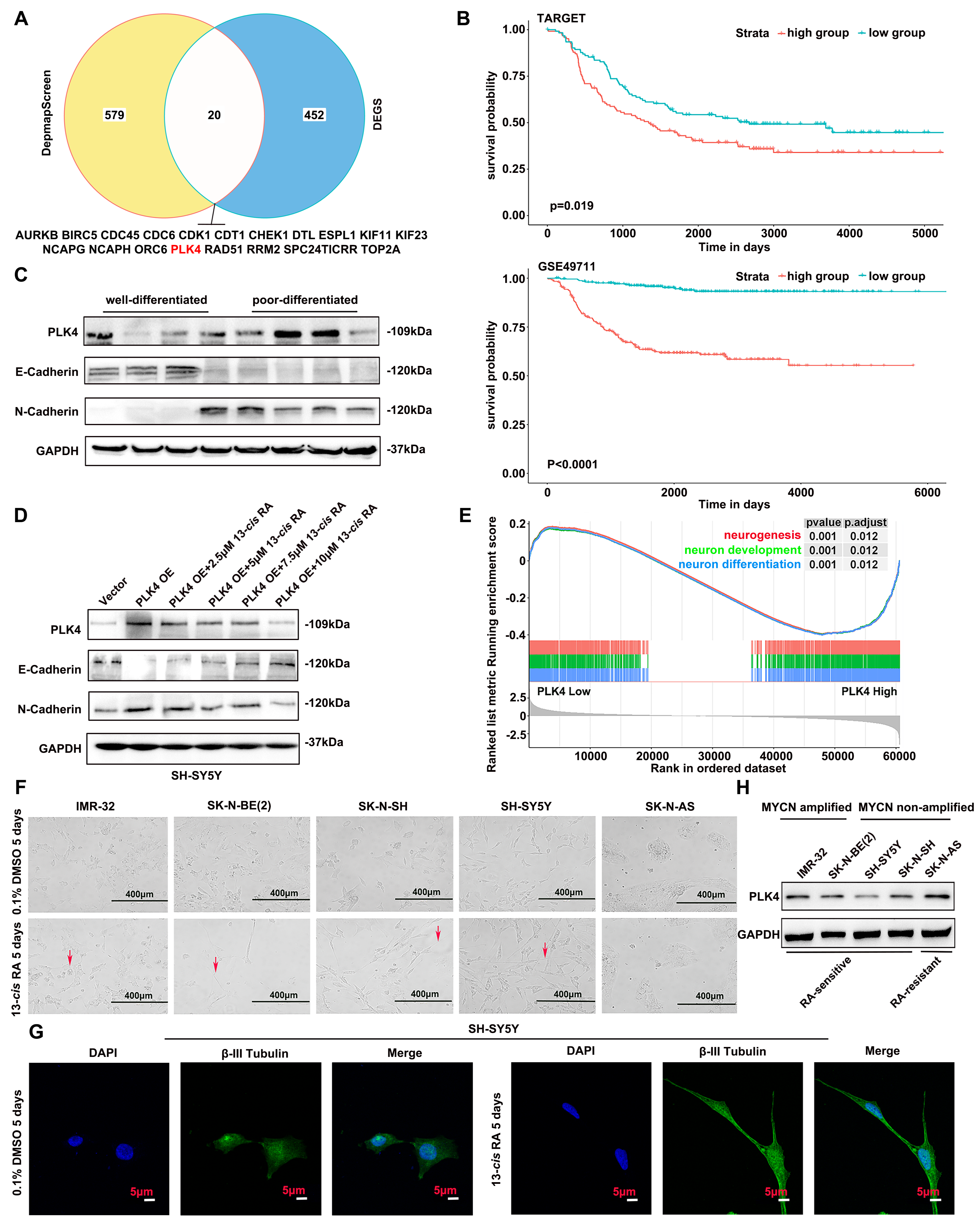

Supplement: Supplementary file 1 — Supplementary figures. [file ijbsv21p4979s1.zip › Supplementary figures/Supplementary figure 1.tif]

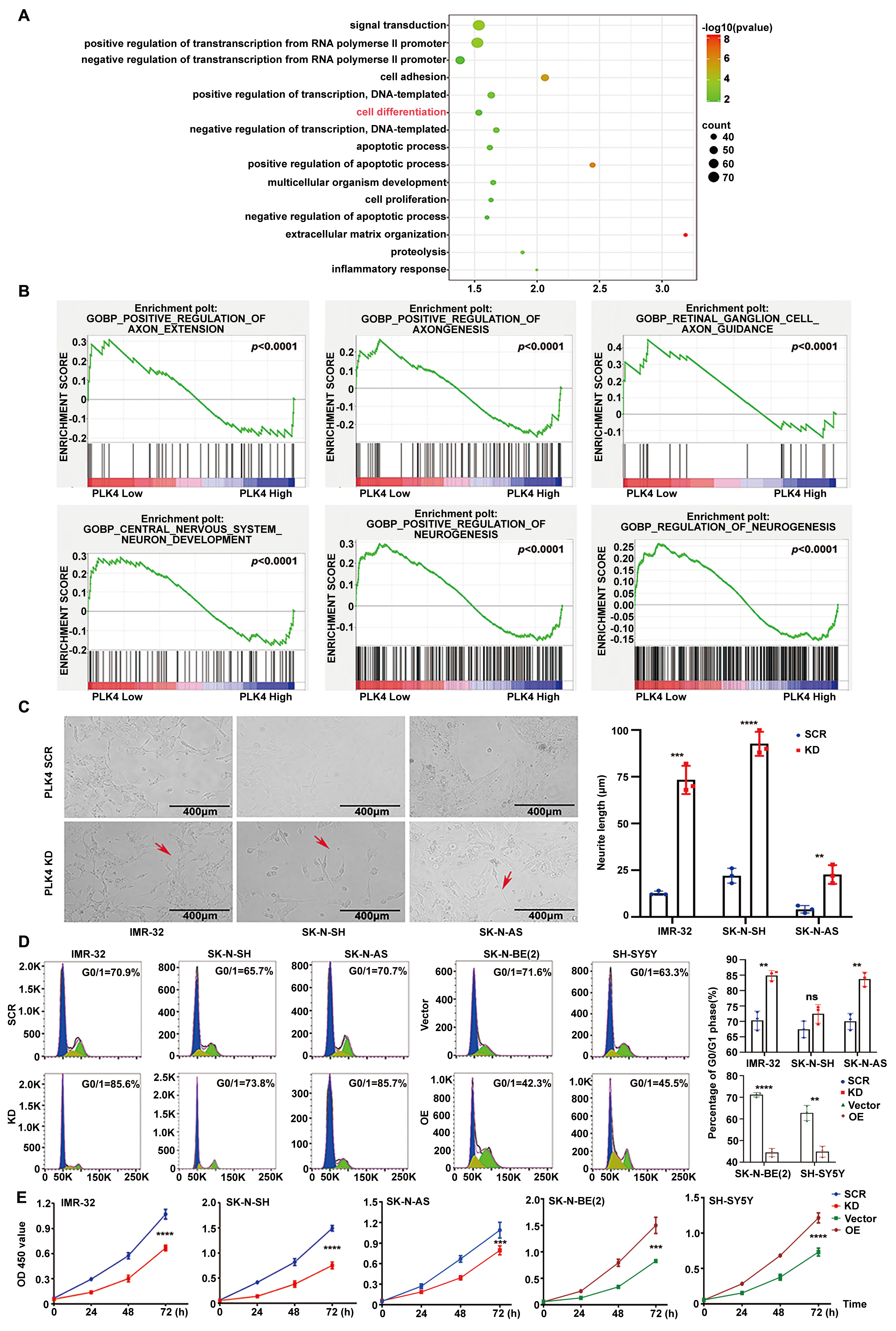

Supplement: Supplementary file 1 — Supplementary figures. [file ijbsv21p4979s1.zip › Supplementary figures/Supplementary figure 2.tif]

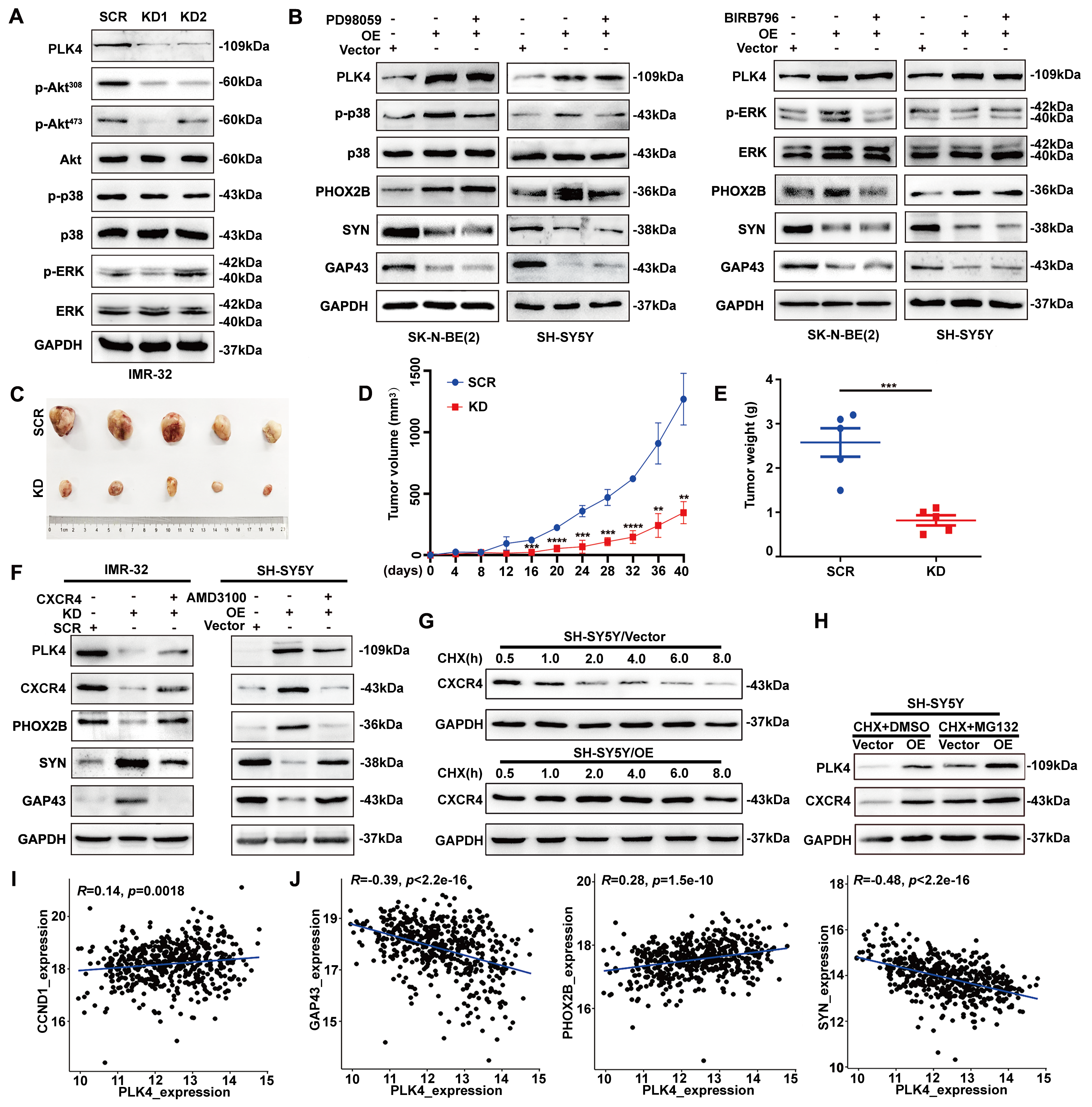

Supplement: Supplementary file 1 — Supplementary figures. [file ijbsv21p4979s1.zip › Supplementary figures/Supplementary figure 3.tif]

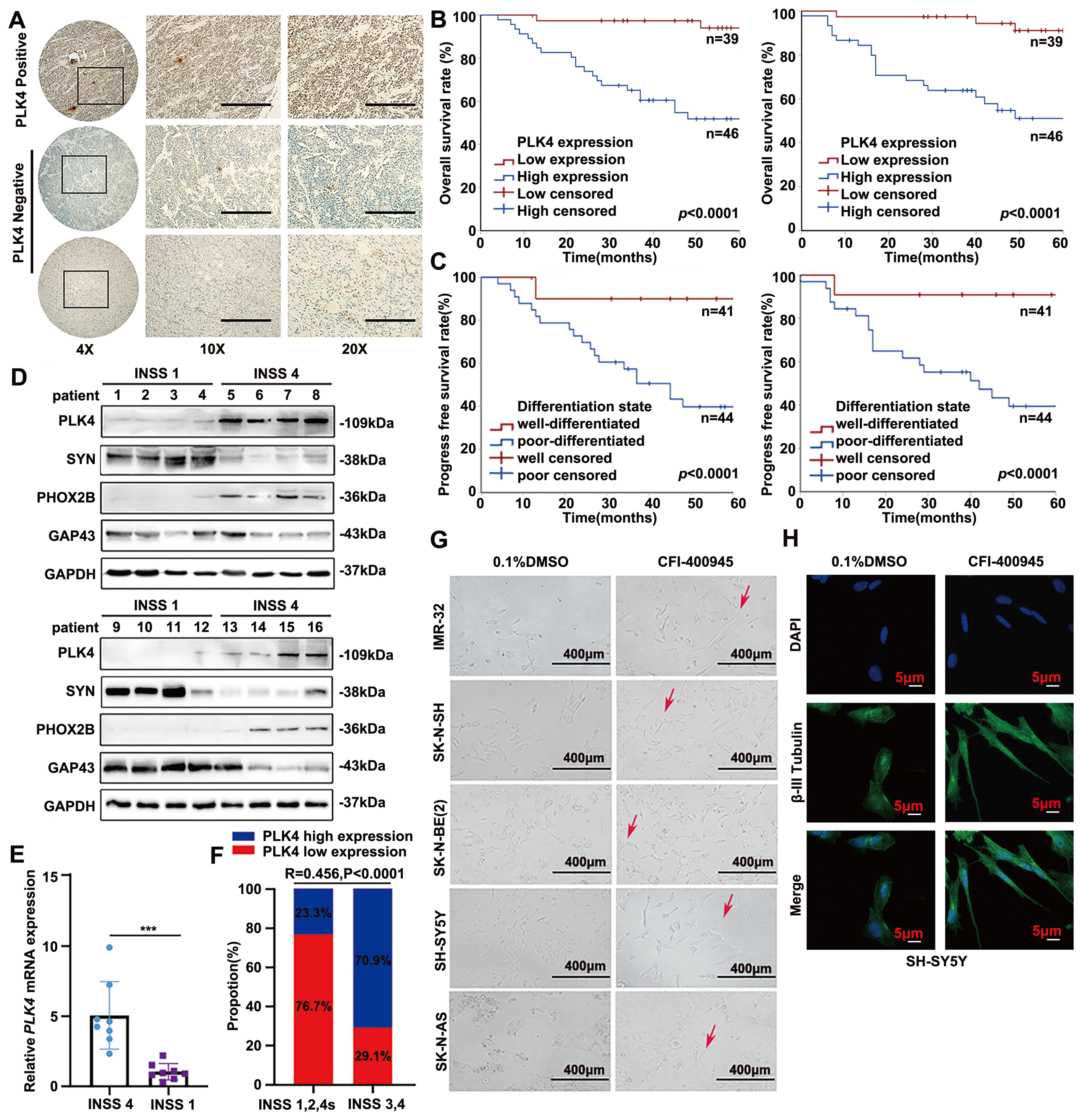

Supplement: Supplementary file 1 — Supplementary figures. [file ijbsv21p4979s1.zip › Supplementary figures/Supplementary figure 4.tif]
